# Long-term prediction of mortality by heart rate turbulence in hemodialysis patients and the impact of diabetes mellitus–a longitudinal observational study

**DOI:** 10.1007/s40620-025-02357-8

**Published:** 2025-07-15

**Authors:** Nora Hannane, Christopher C. Mayer, Julia Matschkal, Felix Bormann, Axel Krieter, Jürgen R. Braun, Claudius Küchle, Lutz Renders, Roman Günthner, Georg Schmidt, Alexander Müller, Siegfried Wassertheurer, Uwe Heemann, Bernhard Haller, Marek Malik, Christoph Schmaderer, Matthias Christoph Braunisch

**Affiliations:** 1 Department of Nephrology, TUM School of Medicine and Health, TUM Universitätsklinikum, Klinikum Rechts Der Isar, Technical University of Munich, Ismaninger Str 22, 81675 Munich, Germany; 2 Center for Health & Bioresources, Medical Signal Analysis, AIT Austrian Institute of Technology GmbH, Vienna, Austria; 3 Dialysis Center Munich Nord, Munich, Germany; 4 Nephrocare Munich East, Munich, Germany; 5 Praxen Dr. Braun, Dialysis Center, Dingolfing, Germany; 6 Klinik Für Innere Medizin I, TUM School of Medicine and Health, TUM Universitätsklinikum, Klinikum Rechts Der Isar, Technical University of Munich, Munich, Germany; 7 TUM School of Medicine and Health, TUM Universitätsklinikum, Klinikum Rechts Der Isar, Institute of AI and Informatics in Medicine, Technical University of Munich, Munich, Germany; 8 National Heart and Lung Institute, Imperial College London, London, UK; 9 Faculty of Medicine, Department of Internal Medicine and Cardiology, Masaryk University, Brno, Czech Republic

**Keywords:** Cardiovascular autonomic dysfunction, Diabetes mellitus, Kidney failure, Heart rate turbulence, Prospective multicenter trial

## Abstract

**Background:**

Diabetes-driven impaired autonomic nervous system function might contribute to increased mortality in hemodialysis patients. Our study aimed to validate heart rate turbulence as a long-term predictor of mortality in this vulnerable cohort.

**Methods:**

Heart rate turbulence is a non-invasive, 24 h electrocardiography-Holter-based assessment of cardiovascular autonomic responses. Hemodialysis patients of the “rISk strAtification in end-stage Renal disease” (ISAR) study, a prospective, multicenter observational study, were followed up for six years. Mortality hazard, and correlations between clinical characteristics and mortality, were assessed using Cox regression models.

**Results:**

Heart rate turbulence measurement at baseline was available in 290 hemodialysis patients, 99 (34%) with diabetes mellitus. In a multivariable analysis, abnormal heart rate turbulence was associated with a 2.1-fold (95% CI: 1.4–3.2; *p* < 0.001) increased risk for all-cause and 3.1-fold (95% CI: 1.5–6.2; *p* = 0.001) increased risk for cardiovascular mortality. The co-occurrence of abnormal heart rate turbulence and diabetes mellitus represented the strongest risk constellation, increasing all-cause mortality risk to a hazard ratio of 5.8 (95% CI: 3.3—10.4; *p* < 0.001) and cardiovascular mortality risk to 6.1 (95% CI: 2.5—15.1; *p* < 0.001). This association with mortality risk remained significant after multivariate adjustment. The interaction term between the two comorbidities indicated an approximately additive effect on mortality risk.

**Conclusions:**

Heart rate turbulence significantly contributed to the prediction of long-term mortality risk in hemodialysis patients. Diabetes mellitus is a major driver of cardiovascular autonomic dysfunction, which plays a crucial role in mortality among dialysis patients. Heart rate turbulence measurement identifies high-risk patients in the dialysis setting, enhancing precision in risk prediction and stratification, and allowing an opportunity for personalized monitoring and prevention.

**Graphical abstract:**

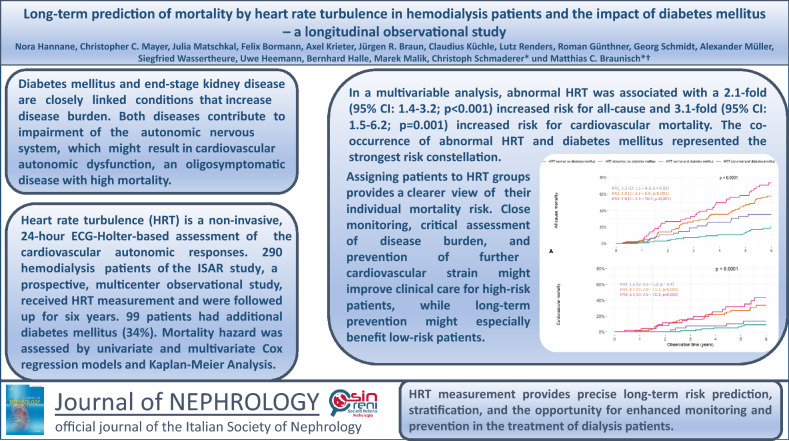

**Supplementary Information:**

The online version contains supplementary material available at 10.1007/s40620-025-02357-8.

## Introduction

Diabetes mellitus and kidney failure are two frequently associated diseases. Nearly 44% of patients in the United States and 24% of patients in Europe have diabetes mellitus as the underlying cause for kidney replacement therapies [1, 2]. Both diabetes mellitus and kidney failure are well-known risk factors for the development of cardiovascular disease (CVD), and their coexistence further exacerbates this risk [3]. In individuals with diabetes mellitus, traditional cardiovascular risk factors such as hypertension, dyslipidemia, obesity, chronic inflammation, and oxidative stress have a higher prevalence and promote the development of atherosclerosis [4]. The further increased risk of CVD in hemodialysis patients can be attributed to the additive effect of dialysis-related risk factors, such as anemia, uremic toxins, electrolyte disturbances, mineral bone disorders, and non-physiological fluid shifts [5].

The non-invasive heart rate turbulence measurement reflects cardiac autonomic nervous responsiveness, which can be assessed in 24-h ECG recordings. Abnormal heart rate turbulence has been associated with an increased risk of cardiac events such as arrhythmias, heart failure, and sudden cardiac death [6]. Heart rate turbulence is a surrogate for the baroreflex and reflects cardiovascular sympathovagal balance. Heart rate turbulence categories have been repeatedly associated with an increased risk of cardiac events in patients with a history of myocardial infarction or heart failure [7, 8].

Cardiovascular autonomic dysfunction is often underdiagnosed due to its minimally symptomatic manifestation. The EURODIAB IDDM Complications study showed a prevalence of autonomic neuropathy in 36% of diabetes type I patients [9]. The study found an association between diabetic retinopathy and albuminuria and the progression of cardiovascular autonomic dysfunction [10]. Pathophysiologic mechanisms involve hyperglycemia, oxidative stress, inflammatory pathways leading to vascular damage, and neuronal toxicity of the autonomous system [11]. As seen in somatic neuropathies, this damage gradually progresses, primarily affecting the vagus nerve, which is mainly responsible for parasympathetic organism functions [12].

We have previously examined heart rate turbulence-based risk prediction in hemodialysis patients with a median follow-up of three years [13]. We identified patterns of heart rate turbulence risk categories in hemodialysis patients similar to those in cardiac disease [13]. We have previously also identified the impact of diabetes mellitus on the turbulence slope, one of the heart rate turbulence components. At present, we have completed the six-year follow-up of our prospective multicenter clinical trial, aimed at examining the long-term predictive value of heart rate turbulence in the kidney failure cohort and the role of diabetes mellitus in risk assessment.

## Materials and methods

### Study population

The investigation utilized six years of follow-up data from the ISAR cohort study. The “rISk strAtification in end-stage Renal disease” (ISAR) study was a prospective observational multicenter study that recruited patients in the greater Munich area (ClinicalTrials.gov; identifier number: NCT01152892) [14]. The detailed study protocol and methods have previously been described [14]. In brief, 17 hemodialysis centers recruited 519 patients from April 2010 to January 2014 based on the following inclusion criteria: written informed consent, age ≥ 18 years, and dialysis vintage ≥ 90 days. Exclusion criteria were pregnancy, ongoing infection or malignancy, and life expectancy of less than 24 months.

### Diabetes mellitus

Baseline variables, as well as the diagnosis of diabetes mellitus, its type, and its association as the primary cause of kidney failure, were collected from medical records. The treatment of diabetes mellitus was assessed in medical records. Comorbidities were assessed using the modified version of the Charlson comorbidity index and cardiovascular risk score for hemodialysis patients [15]. Laboratory workup, including HbA1c, was determined before a midweek dialysis session.

### Study endpoints

The primary and secondary endpoints of cardiovascular mortality and all-cause mortality, respectively, were ascertained based on medical reports or information from the attending physician or next of kin. The endpoints were collected during a follow-up period of six years, and all were validated by the ISAR Endpoint Committee [14].

### Assessment of heart rate turbulence

In each patient, a 24 h-ECG recording was started before a mid-week dialysis session. The collection and processing of the 24 h-ECG recordings have previously been described [13]. In brief, we used the 12-lead ECG Lifecard CF digital Holter recorder of Delmar Reynolds/Spacelabs Healthcare. The evaluation was performed using the corresponding software tool for ECG annotations and RR-interval measurements (Pathfinder, Delmar Reynolds/Spacelabs Healthcare, Nuremberg, Germany; v.9.027). Heart rate turbulence measurement was performed in 390 patients, of whom 290 provided data suitable for analysis (100 patients were excluded because of atrial fibrillation, implanted pacemaker, or other complex arrhythmias). The physiological reaction following a premature ventricular contraction initially led to a transient acceleration in heart rate through vagal withdrawal, followed by a gradual deceleration of heart rate due to parasympathetic restoration and sympathetic-mediated increase of atrial resistance. Turbulence onset of this deceleration and slope of the heart rate variability were calculated if at least five premature ventricular contractions were present [16]. Based on these values, the three heart rate turbulence groups 0, 1, and 2 were calculated as previously described [7, 8].

### Statistics

Categorical data are reported as absolute values and relative frequencies and were compared using the Chi-square test. Continuous variables are presented as mean and standard deviation or, where appropriate, as median and interquartile range. The normal distribution of continuous variables was tested using the Kolmogorov–Smirnov test. A statistical comparison of two groups was done using the *t*-test for independent samples or the Mann–Whitney U test for > 2 groups using analysis of variance (ANOVA) or the Kruskal–Wallis test, as appropriate.

The cumulative mortality rate was estimated by the Kaplan–Meier method and tested for group differences with the log-rank test. Cumulative incidence functions were computed for the endpoints, and cause-specific hazards were compared between groups by the log-rank test (Fine and Gray model). We used the *cmprsk* package in R, which allows us to fit proportional sub-distribution hazard models [17]. Parameters for adjustment were chosen if they were significantly associated with mortality in a univariable Cox proportional hazards regression (baseline characteristics without medications). Only one variable was selected from variables with a high co-linearity (e.g., serum-albumin and creatinine, C-reactive protein and interleukin 6, history of myocardial infarction and coronary heart disease). The final adjusted model included diabetes mellitus and heart rate turbulence, age, creatinine, high-sensitivity C-reactive protein, history of myocardial infarction, peripheral arterial disease, and diastolic blood pressure. In a secondary analysis, other heart rate variability parameters, and  administered medications were tested towards the endpoints. The cardiovascular mortality multivariate model was limited in the number of variables due to the lower event rate (*n* = 50). We therefore tested medications individually within the multivariate model to avoid overfitting (calcium channel blockers or diuretics or statins). All tests were performed two-sided, and a p-value of < 0.05 was considered significant. Analysis was performed using R and R-Studio (R Version 4.2.2 and R Studio Version 2024.04.2 Build 764).

## Results

### Patient characteristics

Overall, 290 hemodialysis patients with heart rate turbulence measurement were followed up for as long as six years. The median follow-up duration was 4.3 years. One hundred-eighteen patients completed the 6-year follow-up, 111 patients died, 41 patients received a kidney transplant, and 20 patients were lost to follow-up due to transfer to other dialysis centers and cities. In 246 patients (85%), hemodiafiltration, and in 44 patients, hemodialysis treatment was delivered. Dialysis was performed thrice weekly in 258 patients (89%); other patients had a lower (*n* = 9) or higher (*n* = 23) frequency. One hundred ninety-one patients (66%) had no diabetes mellitus, while 99 patients (34%) had a documented diagnosis of diabetes mellitus. In comparison to patients without diabetes (*n* = 191), patients with diabetes mellitus (*n* = 99) were significantly older (67 ± 12 vs. 59 ± 16; *p* < 0.001), had a shorter dialysis vintage (35 months [20–58] vs. 55 months [28–93]; *p* < 0.001), higher BMI (27 [24–31] vs. 24 [22–28]; *p* < 0.001), higher HbA1c (6.7%, [6.2–7.6] vs. 5.3% [5.1–5.5]; *p* < 0.001), lower diastolic blood pressure (72 ± 14 mmHg vs. 77 ± 15 mmHg; *p* = 0.011), and lower creatinine (7.4 ± 2.4 mg/dl vs. 9.5 ± 2.7 mg/dl; *p* < 0.001). Patients with diabetes mellitus also had a significantly greater history of myocardial infarction (26% vs. 11%; *p* = 0.001), peripheral arterial disease (28% vs. 14%; *p* = 0.005), cerebrovascular disease (22% vs. 10%; *p* = 0.007), and coronary heart disease (46% vs. 22%; *p* < 0.001) (Table 1).

**Table 1:** Baseline characteristics of the study population stratified by diabetes mellitus status

Parameters	No diabetes mellitus (*n* = 191, 66%)	Diabetes mellitus (*n* = 99, 34%)	*P*
Age (years)	59.3 (± 16.2)	66.9 (± 12.2)	< 0.001
Sex, female	65 (65.0%)	35 (35.0%)	0.90
Body mass index (kg/m^2^)	24.3 (22.2—27.6)	27.1 (23.6—31.3)	< 0.001
Dialysis vintage (months)	55.0 (28.0—93.0)	35.0 (20.0—57.5)	< 0.001
Ultrafiltration rate (mL/h)	451.6 (± 250.8)	543.3 (± 266.5)	0.004
Bodyweight adjusted ultrafiltration rate (mL/h/kg)	6.3 (± 3.6)	6.9 (± 3.8)	0.2
Net ultrafiltration (L)	1.6 (± 1.2)	1.9 (± 1.1)	0.051
Heart rate (bpm)	74.9 (± 10.9)	72.7 (± 11.0)	0.099
Systolic blood pressure (mmHg)	136.1 (± 20.3)	138.4 (± 25.1)	0.41
Diastolic blood pressure (mmHg)	76.6 (± 14.6)	72.1 (± 14.1)	0.011
Kt/V	1.49 (± 0.40)	1.42 (± 0.36)	0.11
Blood urea nitrogen (mg/dL)	62.8 (± 17.3)	62.8 (± 14.8)	0.99
Phosphate (mmol/L)	1.70 (1.40—2.03)	1.68 (1.32—2.10)	0.77
Total calcium (mmol/L)	2.28 (± 0.18)	2.26 (± 0.19)	0.56
Calcium x phosphate (mmol^2^/L^2^)	3.97 (± 1.09)	3.89 (± 1.16)	0.55
Creatinine (mg/dL)	9.5 (± 2.7)	7.4 (± 2.4)	< 0.001
High-sensitivity CRP (mg/dL)	0.34 (0.16—0.81)	0.53 (0.17—0.96)	0.16
Albumin (g/dL)	4.10 (3.84—4.30)	4.00 (3.70—4.20)	0.035
Parathyroid hormone (pg/mL)	268.8 (137—433)	204.2 (103—369)	0.074
Leukocytes (G/L)	6.73 (± 1.97)	7.43 (± 2.17)	0.006
Interleukin-6 (pg/ml)	7.78 (4.2—12.1)	7.57 (5.6—12.2)	0.64
Total cholesterol (mg/dL)	182.0 (± 46.2)	182.3 (± 42.4)	0.96
HbA1c (%)	5.3 (5.1—5.5)	6.7 (6.2—7.6)	< 0.001
Charlson Comorbidity Index (0 to 21)	2.0 (0.0—4.0)	4.0 (2.0—6.0)	< 0.001
Cardiovascular mortality risk score (-11 to 39)	8.0 (3.0—11.0)	12.0 (8.5—15.5)	< 0.001
History of myocardial infarction	21 (11%)	26 (26%)	0.001
Left ventricular hypertrophy	43 (23%)	33 (33%)	0.050
Heart failure	17 (9%)	14 (14%)	0.23
Peripheral artery disease	27 (14%)	28 (28%)	0.005
Hypertension	176 (92%)	95 (96%)	0.32
Coronary heart disease	42 (22%)	46 (46%)	< 0.001
Cerebrovascular disease	19 (10%)	22 (22%)	0.007
Medication			
ß-blocker	112 (60.9%)	72 (39.1%)	0.021
Antihypertensive medication	163 (85%)	95 (96%)	0.005
Diuretics	97 (51%)	73 (74%)	< 0.001
Statins	57 (30%)	47 (48%)	0.004
Mortality			
All-cause mortality events	59 (30.9%)	51 (52%)	
Cardiovascular mortality events	28 (14.7%)	20 (20%)	

Results are presented as mean (± SD) and median (interquartile range) for normally and non-normally distributed data, respectively, and categorical data as total number (percentage)

Kt/V, Urea clearance per time and volume; CRP, C-reactive protein; HbA1c, glycated hemoglobin

^†^P values present the results of group-wise comparisons of patients without diabetes mellitus and with diabetes mellitus by T-test or Mann Whitney U test

### Mortality

During the six-year observation period, a total of 110 patients died. Of these deaths, 48 (44%) were of cardiovascular origin, 32 (39%) were caused by infection, and 30 (26%) had other causes, including cancer (*n* = 6, 6%), termination of dialysis therapy as palliative care (*n* = 6, 6%), or had unknown or unreported causes (*n* = 18, 17%).

Significant risk factors for mortality were higher age, frailty (indicated by lower creatinine, blood urea nitrogen, and albumin), cardiovascular diseases (history of myocardial infarction, coronary heart disease, cerebrovascular disease, peripheral arterial disease), and greater signs of inflammation (high C-reactive protein, high interleukin 6, higher leukocytes), as shown in Table 2.

**Table 2:** Univariate and multivariate Cox regression of risk factors for all-cause and cardiovascular mortality (6 years)

Variable	Univariate		Multivariate	
	Hazard ratio (95% CI)	*P*	Hazard ratio (95% CI)	*P*
All-cause mortality				
Presence of diabetes mellitus	1.8 (1.2—2.6)	0.002	1.1 (0.7 – 1.6)	0.7
Pathological heart rate turbulence	3.4 (2.3—5.1)	< 0.001	2.1 (1.4—3.2)	< 0.001
Diabetes mellitus and pathological heart rate turbulence	5.8 (3.3—10.4)	< 0.001	2.2 (1.2—4.3)	0.01
Age per 1 year	1.1 (1.0—1.1)	< 0.001	1.0 (1.0—1.1)	0.001
Creatinine per 1 mg/dL	0.83 (0.8—0.9)	< 0.001	0.9 (0.8—1.0)	0.07
History of myocardial infarction	2.3 (1.5—3.5)	< 0.001	1.5 (0.9—2.4)	0.02
Coronary heart disease	2.3 (1.6—3.4)	< 0.001	–	–
Cerebral arteriovascular disease	2.3 (1.5—3.5)	< 0.001	–	–
Peripheral arteriovascular disease	2.6 (1.7—3.8)	< 0.001	1.7 (1.1—2.7)	0.02
Blood urea nitrogen per 1 mg/dL	0.97 (0.96—0.98)	< 0.001	-	-
High-sensitivity CRP	1.2 (1.1—1.4)	0.003	1.1 (1.0—1.3)	0.07
Interleukin 6	1.0 (1.0—1.1)	< 0.001	–	–
Serum albumin	0.3 (0.2—0.5)	< 0.001	–	–
Charlson comorbidity index	1.2 (1.2—1.3)	< 0.001	–	–
Heart failure	1.5 (0.9 – 2.6)	0.15	–	–
Mean Heart rate	0.6 (0.4 – 0.7)	< 0.001	–	–
Systolic blood pressure	1.0 (0.9 – 1.0)	0.5	–	–
Diastolic blood pressure	0.98 (0.96 – 0.99)	0.001	–	–
Cardiovascular mortality				
Presence of diabetes mellitus	1.5 (0.8—2.6)	0.18	0.8 (0.4—1.6)	0.6
Pathological heart rate turbulence	4.4 (2.3—8.4)	< 0.001	2.9 (1.5—5.8)	0.002
Diabetes mellitus and pathological heart rate turbulence	6.1 (2.5—15.1)	< 0.001	2.9 (1.1—7.9)	0.05
Age per 1 year	1.0 (1.0—1.1)	< 0.001	1.0 (1.0—1.1)	0.2
Creatinine per 1 mg/dL	0.9 (0.8—0.9)	0.008	0.9 (0.8—1.1)	0.9
History of myocardial infarction	3.4 (1.9—6.1)	< 0.001	2.7 (1.3—5.8)	0.006
Coronary heart disease	3.0 (1.7—5.2)	< 0.001	–	–
Peripheral arteriovascular disease	2.2 (1.2—4.1)	0.01	1.4 (0.7—2.8)	0.3
Blood urea nitrogen per 1 mg/dL	0.97 (0.96—0.99)	0.03	–	–
Charlson comorbidity index	1.3 (1.2—1.4)	< 0.001	–	–
Cardiovascular risk-score	1.1 (1.0—1.1)	0.007	–	–
Leukocytes	1.2 (1.0—1.3)	0.04	–	–
Heart failure	2.2 (1.1—4.5)	0.03	–	–
Mean heart rate	0.6 (0.4 – 0.9)	0.02	–	–
Systolic blood pressure	1.0 (0.9 – 1.0)	0.7	–	–
Diastolic blood pressure	0.99 (0.97 – 1.0)	0.5	–	–

Baseline characteristics (without medication) were examined through univariate Cox regression regarding their association with all-cause and cardiovascular mortality. Significant variables are shown in this table, as well as functional relevant cardiovascular factors. For multivariate testing, factors were summarized as age, creatinine, history of myocardial infarction, peripheral arterial disease, and high-sensitivity CRP in avoidance co-linearity (see material and methods). CI, confidence interval; CRP, C-reactive protein

### Long-term association of heart rate turbulence and mortality

**Fig. 1:**
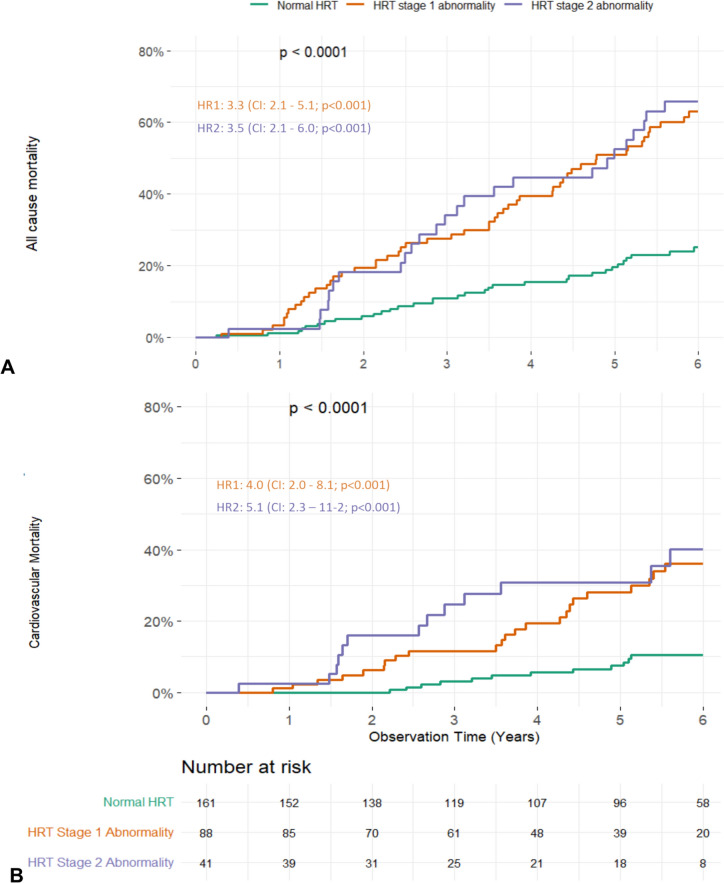
Cumulative six-year mortality curves for all-cause (**A**) and cardiovascular mortality (**B**) stratified by heart rate turbulence (HRT) groups. A strong and significant increase in all-cause and cardiovascular six-year mortality was observed in patients with pathological HRT. Patients in HRT group 1 showed a 3.3-fold (HR, 95% CI: 2.1–5.1; *p* < 0.001) increase, patients in HRT group 2 showed a 3.5-fold (HR, 95% CI: 2.1–6.0; *p* < 0.001) increase of all-cause mortality risk compared to patients with normal HRT measurement. For cardiovascular mortality, a 4.0-fold (HR, 95% CI: 2.0–8.1; *p* < 0.001) increased risk was observed in the HRT group 1, and a 5.1-fold (HR, 95% CI: 2.3–11.2; *p* < 0.001) increased risk in the HRT group 2 compared to patients with normal HRT. Interestingly, we did not observe further discrimination between HRT groups 1 and 2 after six years. Therefore, we dichotomized the HRT groups into pathological and normal HRT for additional analyses

Heart rate turbulence measurement comprises two parameters—turbulence onset and slope – which reflect autonomic balance following a premature ventricular contraction. Patients with a pathological value in one of the two parameters were classified as heart rate turbulence group 1, and those with abnormal values in both turbulence onset and turbulence slope were classified as heart rate turbulence group 2, indicating more severe autonomic dysregulation. Our 6-year observational data showed mortality differences between patients with normal heart rate turbulence values and heart rate turbulence abnormality group 1 or 2 patients. During the first 4 years of the observation period, stratification among all three groups was seen. Interestingly, there were no significant differences between heart rate turbulence abnormality group 1 patients and heart rate turbulence abnormality group 2 patients following these 4 years concerning 6-year all-cause and cardiovascular mortality (Fig. 1). Therefore, for the long-term mortality analysis we dichotomized patients with normal and abnormal values (i.e., heart rate turbulence category 0 vs. heart rate turbulence categories 1 and 2 combined). In the univariate Cox regression, an abnormal heart rate turbulence showed a 3.4-fold (Hazard Ratio [HR], 95% confidence interval [CI]: 2.3–5.1; *p* < 0.001) increase of all-cause mortality risk and a 4.4-fold (HR, 95% CI: 2.3–8.4; *p* < 0.001) increase of cardiovascular mortality (Table 2). In total, the estimated probability for cardiovascular mortality was 10% for patients with normal heart rate turbulence compared to 36% and 40% among patients with heart rate turbulence in group 1 and group 2, respectively. This difference remained significant after multivariable adjustment for further covariates with a 2.1-fold (HR, 95% CI: 1.4–3.2; *p* < 0.001) increase of all-cause and a 3.1-fold (HR, 95% CI: 1.5–6.2; *p* = 0.001) increase of cardiovascular mortality risk.

In the next step, we included administered medications into our multivariate model. In univariate testing diuretics and statins showed a significant association with all-cause and cardiovascular mortality, while calcium channel blockers showed a significant association with cardiovascular mortality alone. After multivariate adjustment, only calcium channel blockers showed a significantly lower risk for cardiovascular mortality. In this secondary analysis, heart rate turbulence remained a robust significant risk factor for all-cause and cardiovascular mortality when adjusted for medical treatments (Supplementary Table 1).

In a secondary analysis, we investigated the association of other heart rate variability parameters with all-cause and cardiovascular mortality. Several parameters showed a univariate significant risk association with mortality. In multivariate analysis, the following parameters were associated with all-cause mortality: Heart rate variability triangular index (HRVI), Standard deviation of the average NN intervals (SDANN), Total power, Ultra-low frequency, and Deceleration capacity category 2. Standard deviation of all NN intervals (SDNN), Standard deviation of average NN intervals (SDANN), Total power, and ultra low frequeny were associated with cardiovascular mortality (Supplementary Table 2).

### Association of heart rate turbulence and diabetes mellitus with mortality

**Fig. 2:**
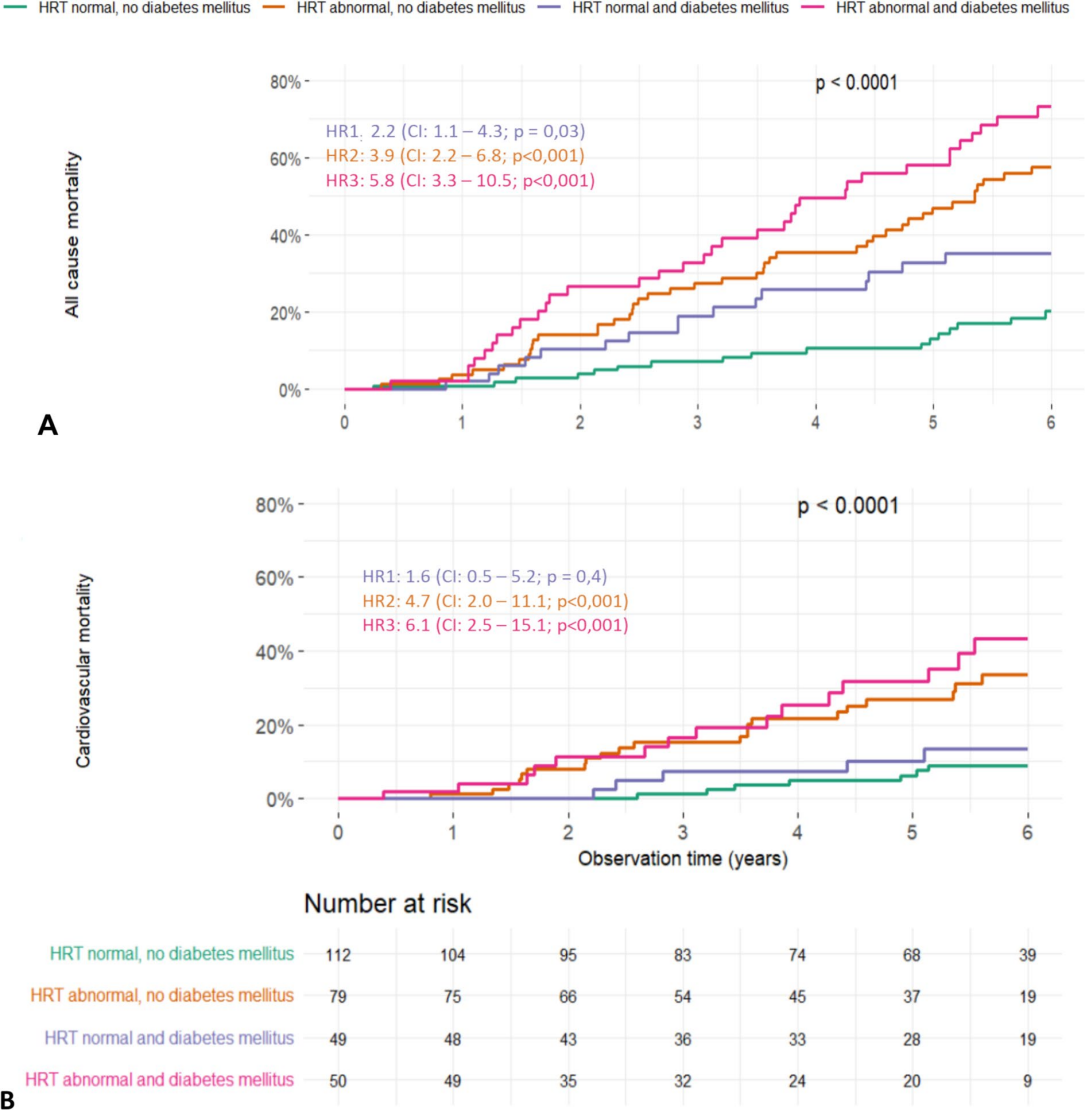
Cumulative six-year mortality curves stratified by a combination of diabetes mellitus status and heart rate turbulence. We built four subgroups by dichotomizing patients with and without diabetes mellitus and pathological vs normal heart rate turbulence (HRT) and stratified these groups towards all-cause (**A**) and cardiovascular mortality (**B**). The lowest mortality risk was observed in patients without diabetes mellitus and with normal HRT measurement. The highest mortality risk was seen in diabetes patients with pathological HRT measurement with a univariate 5.8-fold (HR, 95% CI: 3.3–10.5; *p* < 0.001) increase of all-cause and 6.1-fold (HR, 95% CI: 2.5–15.1; *p* < 0.001) increase of cardiovascular mortality. After adjustment, this effect remained significant with a 2.1-fold (HR, 95% CI: 1.2–3.9; *p* = 0.01) increase in all-cause mortality and 3.0-fold (HR, 95% CI: 1.1–8.5; *p* = 0.03) of cardiovascular mortality

We also examined the interaction of heart rate turbulence alterations in patients with diabetes mellitus. The co-occurrence of abnormal heart rate turbulence and diabetes mellitus represented the strongest risk constellation, increasing all-cause mortality risk to a hazard ratio of 5.8 (95% CI: 3.3—10.4; *p* < 0.001) and cardiovascular mortality risk to 6.1 (95% CI: 2.5—15.1; *p* < 0.001) (Table 2). This association with mortality risk remained significant after multivariate adjustment. The interaction term between heart rate turbulence pathologies and diabetes mellitus in the Cox regression analysis was 0.7 (95% CI 0.3–1.6; *p* = 0.4) for all-cause mortality and was not significant. A ratio below 1 and an insignificant p-value suggest that the combined effect of these two variables on mortality is primarily additive, with no further interaction between the two pathologies. The additive effect of the two pathologies can also be retraced in the Kaplan–Meier curves, where the hazard ratio of the combined pathologies represents nearly the addition of the single risk curves of diabetes mellitus and pathological heart rate turbulence (Fig. 2). Furthermore, the prevalence of heart rate turbulence pathologies was higher in patients with diabetes mellitus than in those without (51% versus 41%).

## Discussion

Identification of risk factors and mortality prediction remains challenging in the vulnerable population of dialysis patients. We observed that the combination of diabetes mellitus and abnormal heart rate turbulence showed a very high unadjusted mortality rate compared to abnormal heart rate turbulence or diabetes mellitus alone, and this high risk remained significant after adjustment for other risk factors.

The cardiovascular autonomic nervous system is one of the most important regulators of both heart rate and blood pressure [18]. Diabetes mellitus and kidney failure can harm the sympathetic and parasympathetic nerves, leading to their dysfunction [19]. Prior studies identified a high prevalence of cardiovascular autonomic dysfunction in diabetes patients, especially in those with type I diabetes mellitus and long-term disease [11]. Pathophysiological mechanisms such as hyperglycemia, inflammation, and atherosclerosis lead to microvascular damage of the nerves in a length-dependent manner, thus, in particular, affecting the vagal nerve [12]. The resulting disease is often oligosymptomatic and consequently underdiagnosed because of nonspecific manifestations such as exercise intolerance, tachycardia, vertigo, and fainting. The assessment of heart rate turbulence quantifies the interaction between initial sympathetic activity caused by parasympathetic withdrawal in response to hemodynamic compromise due to an extrasystole, followed by a gradual reinstitution of vagal activity. Impaired heart rate turbulence represents a dysfunction of the autonomic cardiovascular system [20] and was observed to be a highly significant risk factor in our hemodialysis patients. The assessment of heart rate turbulence enabled a strong prediction of six-year mortality, which was also valid after adjustment for other well-known risk factors. Interestingly, the classification of abnormal heart rate turbulence severity into the standard groups of heart rate turbulence 1 and 2 did not lead to further significant risk increases in our long-term data, after exceeding an observation period of 4 years. Follow-ups regarding mortality risk stratification in previous studies in patients after myocardial infarction or heart failure only rarely exceeded this observation period [21, 22]. We hypothesize that due to continuous hemodialysis treatment, impairment of the autonomic nervous system might progress, leading to the loss of differences in cardiovascular mortality between heart rate turbulence categories 1 and 2 after three years of follow-up. This impairment might be more substantial than in patients with congestive heart failure, where a separation into the three heart rate turbulence groups can be observed for up to four years [23]. Nevertheless, the distinction between normal and abnormal heart rate turbulence remained a strong predictor of all-cause and cardiovascular mortality, underlining the method's robustness even with a prolonged 6-year follow-up. Therefore, we can confirm that heart rate turbulence assessment is suitable for long-term risk prediction in dialysis patients. Categorization into two severity categories of heart rate turbulence might, thus, be primarily of importance in short-term risk prediction models of cardiovascular mortality in hemodialysis patients [13].

The impact of heart rate turbulence pathologies on all-cause mortality represents an interesting finding. The influence of cardiovascular autonomic dysfunction on all-cause mortality might reflect a poorer ability to adapt to changing demands. An interaction involving the cholinergic anti-inflammatory pathway via the vagus nerve might have a negative impact on infection-associated events [24]. This may induce a general dysregulation of the immune system and increase the risk of severe complications in the context of infections. Also, generally increased frailty of patients, e.g., by orthostatic dysregulation complicating mobility, difficult feeding in the case of other autonomic regulatory disorders, and reduced resilience, appears conceivable.

Interestingly, additional heart rate variability parameters were also significantly associated with the endpoints. While our primary focus was on heart rate turbulence — based on earlier findings showing its exclusive predictive value for cardiovascular mortality after three years [13] — we now observe, in this extended six-year follow-up, that single indices such as heart rate variability index, SDNN, SDANN, total power, ultra low frequency, and deceleration capacity are also linked to mortality. Notably, some measures like heart rate variability index and deceleration capacity were predictive only for all-cause mortality, likely due to the higher event rate. The heterogeneous associations of individual heart rate variability parameters with varying mortality risks complicate their general clinical application—especially when contrasted with the consistent predictive value and clear risk stratification offered by heart rate turbulence.

Diabetes mellitus was associated with increased mortality in our cohort; however, this association did not remain significant after adjustment for age and other comorbidities. In clinical practice, evaluating heart rate turbulence status in dialysis patients with diabetes provides a more accurate risk assessment than considering diabetes alone. The interaction analysis did not show further interactions between the two comorbidities. The increased mortality risk must therefore be seen as the sum of the individual risks of each disease (additive effect).

In pursuing the goal of improving care and risk assessment of dialysis patients, the strong increase in all-cause mortality remains a major challenge. A deeper understanding of cardiovascular disease progression showed the importance of metabolic and inflammatory processes [25]. Understanding the spectrum of cardiovascular diseases is substantially enhanced when considering metabolic and inflammatory processes. These processes' mechanisms and interactions challenge assessing and mitigating cardiovascular risks [26]. The interaction analysis of heart rate turbulence and diabetes mellitus indicated that the effects of both parameters are mainly additive.

The identification of high-risk dialysis patients (as reflected by heart rate turbulence) may increase safety and improve treatment. However, no interventional trials have been carried out based on heart rate turbulence risk stratification to prevent all-cause and cardiovascular mortality. In high-risk individuals, it might be worthwhile to pay special attention to known risk factors that burden the cardiovascular system, especially in the context of cardiovascular autonomic neuropathy, such as avoidance of intradialytic hypotension, volume management, management of heart failure, or anemia [27, 28]. It has been reported that patients with heart rate turbulence pathology (category 2) who have suffered a myocardial infarction may benefit from an implantable cardioverter-defibrillator [8, 22]. This is one of the first interventions resulting from heart rate turbulence assessment to reduce the associated mortality risks, a procedure that has not yet been tested in other high-risk patients with heart rate turbulence pathologies. On the other hand, patients with physiological heart rate turbulence values (with or without diabetes) have a relatively moderate risk profile for all-cause and cardiovascular mortality, which also represents an important increase of available information These patients may particularly benefit from long-term preventive measures such as reduction of cardiovascular risk factors by promoting a healthy lifestyle, optimized treatment of comorbidities such as diabetes mellitus and dialysis-related risk factors (hyperphosphatemia, electrolyte dysregulation, excessive volume shifts) to prevent the development of cardiovascular autonomic neuropathy [29]. Therefore, understanding the risk profile of patients with heart rate turbulence pathologies can support decision-making regarding diagnostic procedures, hospitalization, treatment options, and the interpretation of disease burden [30].

The study's limitations mainly reflect the sample size, the number of cardiovascular events, and specific subgroup analyses. Additionally, clinical information was based on medical records, which might have been influenced by the interpretation of the collecting physician and the quality of the documentation. The study focused on assessing heart rate turbulence and heart rate variability parameters in dialysis patients; other important aspects of cardiovascular dysregulation, such as intradialytic hypotension, were not systematically assessed, representing a limitation due to potential confounding or interacting effects. Also, we did not assess the T-wave alternans parameter, and left-ventricular ejection fraction was only available in a fraction of the patients (*n* = 44), not allowing further analysis of its risk association and interaction with heart rate turbulence pathologies. Finally, the statistical analysis was not defined prospectively. Further studies are needed to investigate morbidity events, enabling a more targeted approach to disease prevention.

## Conclusion

The non-invasive assessment of heart rate turbulence provides valuable insights into the prognosis and may improve the management of hemodialysis patients. In our prospective study, pathological heart rate turbulence was significantly associated with long-term mortality in multivariable analysis. Moreover, in pateints living with diabetes mellitus, adding the characteristics of heart rate turbulence improved risk estimation, compared to diabetic comorbidity alone. Increased attention to cardiovascular autonomic dysfunction may provide an opportunity for individualized approaches and ultimately improve care for dialysis patients.

## Supplementary Information

Below is the link to the electronic supplementary material.

Supplementary file1 (DOCX 33 KB)

## Data Availability

Matthias Christoph Braunisch, Nora Hannane and Christoph Schmaderer had full access to the data in the study and take responsibility for the integrity of the data and the accuracy of the data analysis. The patient data will not generally be published to respect data protection and ethical requirements as fixed in the written informed consent. Information and Data will be shared on reasonable request. We strive for better anonymization and broader informed consent of patients to improve data transparency in the future.
