# Supplementary material for: Long-term prediction of mortality by heart rate turbulence in hemodialysis patients and the impact of diabetes mellitus–a longitudinal observational study
Source: J Nephrol. 2025 Jul 15;38(8):2261–72. doi: 10.1007/s40620-025-02357-8 (PMC12630279; doi:10.1007/s40620-025-02357-8)
Supplement: Supplementary file 1 — Supplementary file1 (DOCX 33 KB) [file 40620_2025_2357_MOESM1_ESM.docx]

**Supplementary Material**

**Long-term prediction of mortality by heart rate turbulence in hemodialysis patients and the impact of diabetes mellitus**

**– a longitudinal observational study**

Nora Hannane^1^, Christopher C. Mayer^2^, Julia Matschkal^1^, Felix Bormann^3^, Axel Krieter^4^, Jürgen R. Braun^5^, Claudius Küchle^1^, Lutz Renders^1^, Roman Günthner^1^, Georg Schmidt^6^, Alexander Müller^6^, Siegfried Wassertheurer^2^, Uwe Heemann^1^, Bernhard Haller^7^, Marek Malik^8,9^, Christoph Schmaderer^1^* und Matthias C. Braunisch^1^*^†^

^1^ TUM School of Medicine and Health, TUM Universitätsklinikum, Klinikum Rechts der Isar, Department of Nephrology, Technical University of Munich, Munich, Germany

^2^ Center for Health & Bioresources, Medical Signal Analysis, AIT Austrian Institute of Technology GmbH, Vienna, Austria

^3^ Dialysis Center Munich Nord, Munich, Germany

^4^ Nephrocare Munich East, Munich, Germany

^5^ Praxen Dr. Braun, Dialysis Center, Dingolfing, Germany

^6^ TUM School of Medicine and Health, TUM Universitätsklinikum, Klinikum rechts der Isar, Klinik für Innere Medizin I, Klinikum Rechts der Isar, Technical University of Munich, Munich, Germany

^7^ TUM School of Medicine and Health, TUM Universitätsklinikum, Klinikum rechts der Isar, Institute of AI and Informatics in Medicine, Technical University of Munich, Munich, Germany

^8^ National Heart and Lung Institute, Imperial College London, London, United Kingdom

^9^ Faculty of Medicine, Department of Internal Medicine and Cardiology, Masaryk University, Brno, Czech Republic

*These authors contributed equally

| **Supplementary Table 1:** Univariate and multivariate Cox regression of significant baseline characteristics and medication and the association with all-cause and cardiovascular mortality (6 years).  **All-cause mortality** | | | | |
| --- | --- | --- | --- | --- |
| **Variable** | **Univariate** | | **All- cause mortality multivariate Model*** | |
|  | **Hazard ratio**  **(95% CI)** | ***P*** | **Hazard ratio (95% CI)** | ***P*** |
| Presence of diabetes mellitus | 1.8 (1.2 - 2.6) | 0.002 | 1.1 (0.5 – 2.3) | 0.8 |
| Pathological Heart rate turbulence | 3.4 (2.3 - 5.1) | <0.001 | 2.3 (1.3 – 4.1) | 0.007 |
| Diabetes mellitus and pathological HRT | 5.8 (3.3 - 10.4) | <0.001 | 2.3 (1.2 – 4.4) | 0.01 |
| Age per 1 year | 1.1 (1.0 - 1.1) | <0.001 | 1.0 (1.0 – 1.0) | 0.001 |
| Creatinine per 1 mg/dL | 0.83 (0.8 - 0.9) | <0.001 | 0.9 (0.8 – 1.0) | 0.02 |
| History of myocardial infarction | 2.3 (1.5 - 3.5) | <0.001 | 2.0 (1.2 – 3.3) | 0.008 |
| Peripheral arteriovascular disease | 2.6 (1.7 - 3.8) | <0.001 | 2.1 (1.4 – 3.3) | 0.004 |
| High-sensitivity CRP | 1.2 (1.1 - 1.4) | 0.003 | 1.1 (1.0 – 1.3) | 0.08 |
| ACE inhibitors | 1.0 (0.7 – 1.5) | 1.0 | - | - |
| AT1-receptor blockers | 0.8 (0.5 – 1.3) | 0.4 | - | - |
| Calcium-channel blockers | 0.7 (0.5 – 1.0) | 0.06 | - | - |
| ß- blockers | 1.0 (1.0 – 1.5) | 0.9 | - | - |
| Diuretics | 1.7 (1.1 – 2.6) | 0.009 | 1.0 (0.6 – 1.6) | 0.9 |
| Statins | 1.5 (1.0 – 2.1) | 0.046 | 0.7 (0.5 – 1.1) | 0.1 |
| **Cardiovascular mortality** | | | | |
| **Variable** | **Univariate** | **Cardiovascular mortality multivariate Model^#^** | | |
|  | **Hazard ratio**  **(95% CI)** | ***P*** | **Hazard ratio (95% CI)** | P |
| Presence of Diabetes mellitus | 1.5 (0.8 - 2.6) | 0.18 | 1.0 (0.3 – 3.2) | 0.9 |
| Pathological Heart rate turbulence | 4.4 (2.3 - 8.4) | <0.001 | 3.2 (1.3 – 7.9) | 0.01 |
| Diabetes mellitus and pathological HRT | 6.1 (2.5 - 15.1) | <0.001 | 2.9 (1.1 – 7.9) | 0.04 |
| Age per 1 year | 1.0 (1.0 - 1.1) | <0.001 | 1.0 (1.0 – 1.0) | 0.2 |
| Creatinine per 1 mg/dL | 0.9 (0.8 - 0.9) | 0.008 | 0.9 (0.8 – 1.1) | 0.3 |
| History of myocardial infarction | 3.4 (1.9 - 6.1) | <0.001 | 3.0 (1.5 – 5.9) | 0.002 |
| Peripheral arteriovascular disease | 2.2 (1.2 - 4.1) | 0.01 | 1.7 (0.9 – 3.3) | 0.1 |
| ACE inhibitors | 0.91 (0.5 – 1.6) | 0.8 | - | - |
| AT1-receptor blockers | 0.78 (0.4 – 1.6) | 0.5 | - | - |
| ß- blockers | 1.25 (0.7 – 2.3) | 0.5 | - | - |
| Calcium-channel blockers | 0.46 (0.2 – 0.8) | 0.02 | 0.5 (0.2 – 0.9) | 0.03 |
| Diuretics | 2.38 (1.2 – 4.7) | 0.01 | 2.1 (1.0 – 4.6) | 0.06 |
| Statins | 1.99 (1.1 – 3.5) | 0.02 | 0.9 (0.5 – 1.7) | 0.7 |
| Univariate and multivariate Cox regression analysis of significant baseline characteristics and medication. Similar to the primary analysis, the all-cause multivariate model* of the secondary analysis included diabetes mellitus, heart rate turbulence (HRT), diabetes and pathological HRT, age, creatinine, history of myocardial infarction, peripheral arteriovascular disease and hsCRP in addition to the univariate significant medication of diuretics and statins. The cardiovascular mortality multivariate model^#^ of the secondary analysis was constrained by the low event rate (n=50), limiting the number of variables that could be included. The model therefore comprised diabetes mellitus, heart rate turbulence (HRT), diabetes and pathological HRT, age, creatinine, history of myocardial infarction, peripheral arteriovascular disease, and univariate significant medication included individually within the model (calcium channel blockers or diuretics or statins). | | | | |

| **Supplementary Table 2:** Univariate and multivariate Cox regression of HRV parameters and the association with all-cause and cardiovascular mortality (6 years).  **All-cause mortality** | | | | | | | | | |
| --- | --- | --- | --- | --- | --- | --- | --- | --- | --- |
| **Variable** | **Univariate** | | | | **Multivariate*** | | | | |
|  | **Hazard ratio**  **(95% CI)** | ***P*** | **Hazard ratio (95% CI)** | | | ***P*** | |  |  |
| HRVI | 0.9 (0.9 – 1.0) | <0.001 | 1.0 (0.9 – 1.0) | | | 0.005 | |  |  |
| SDNN per 100 ms | 0.2 (0.1 – 0.5) | <0.001 | 0.4 (0.2 – 1.0) | | | 0.3 | |  |  |
| SDANN per 100 ms | 0.2 (0.1 – 0.5) | <0.001 | 0.4 (0.2 – 0.9) | | | 0.03 | |  |  |
| TINN per 100 ms | 0.7 (0.6 – 0.8) | <0.001 | 0.8 (0.7 – 1.0) | | | 0.02 | |  |  |
| RMSSD per 100 ms | 1.2 (0.1 – 11.4) | 0.9 | 1.0 (0.1 – 12.2) | | | 0.9 | |  |  |
| PNN50 per 10% | 1.1 (0.7 – 1.7) | 0.6 | 1.0 (0.6 – 1.7) | | | 0.9 | |  |  |
| Total Power ln(ms^2^) | 0.6 (0.4 – 0.7) | <0.001 | 0.7 (0.5 – 0.9) | | | 0.02 | |  |  |
| ULF ln(ms^2^) | 0.5 (0.4 – 0.7) | <0.001 | 0.7 (0.5 – 0.9) | | | 0.08 | |  |  |
| VLF ln(ms^2^) | 0.7 (0.5 – 0.8) | <0.001 | 0.8 (0.6 – 1.1) | | | 0.2 | |  |  |
| LF ln(ms^2^) | 0.8 (0.7 – 1.0) | 0.04 | 0.9 (0.8 – 1.2) | | | 0.5 | |  |  |
| HF ln(ms^2^) | 1.1 (09 – 1.3) | 0.5 | 1.0 (0.8 – 1.2) | | | 0.8 | |  |  |
| LF/HF ln | 0.5 (0.4 – 0.7) | <0.001 | 0.8 (0.6 – 1.1) | | | 0.2 | |  |  |
| DC category 1 vs. 0 | 0.4 (0.2 – 0.8) | 0.007 | 0.7 (0.3 – 1.6) | | | 0.4 | |  |  |
| DC category 2 vs. 0 | 0.4 (0.3 – 0.6) | <0.001 | 0.6 (0.4 – 1.0) | | | 0.04 | |  |  |
| AC ms | 1.1 (1.0 – 1.2) | 0.02 | 1.0 (1.0 – 1.1) | | | 0.3 | |  |  |
| **Cardiovascular mortality** | | | | | | | | | |
| **Variable** | **Univariate** | **Multivariate^#^** | | | | | | |  |
|  | **Hazard ratio**  **(95% CI)** | ***P*** | | **Hazard ratio (95% CI)** | | | ***P*** | |  |
| HRVI | 0.9 (0.9 - 1.0) | 0.006 | | 1.0 (0.9 - 1.0) | | | 0.08 | |  |
| SDNN per 100 ms | 0.2 (0.1 – 0.6) | 0.005 | | 0.3 (0.1 – 0.9) | | | 0.03 | |  |
| SDANN per 100 ms | 0.2 (0.1 – 0.6) | 0.004 | | 0.2 (0.1 – 0.8) | | | 0.02 | |  |
| TINN per 100 ms | 0.7 (0.6 – 1.0) | 0.02 | | 0.8 (0.7 – 1.1) | | | 0.2 | |  |
| RMSSD per 100 ms | 1.2 (0.1 – 3.5) | 0.9 | | 1.5 (0.1 – 49) | | | 0.8 | |  |
| PNN50 per 10% | 1.0 (0.6 – 2.0) | 0.8 | | 1.1 (0.5 – 2.1) | | | 0.8 | |  |
| Total Power ln(ms^2^) | 0.5 (0.3 – 0.8) | 0.003 | | 0.6 (0.4 – 0.9) | | | 0.03 | |  |
| ULF ln(ms^2^) | 0.5 (0.3 – 0.8) | <0.001 | | 0.5 (0.3 – 0.9) | | | 0.01 | |  |
| VLF ln(ms^2^) | 0.7 (0.5 – 1.0) | 0.07 | | 0.9 (0.6 – 1.3) | | | 0.4 | |  |
| LF ln(ms^2^) | 0.9 (0.6 – 1.1) | 0.3 | | 1.0 (0.7 – 1.3) | | | 0.8 | |  |
| HF ln(ms^2^) | 1.0 (0.8 – 1.3) | 0.9 | | 1.0 (0.7 – 1.3) | | | 0.7 | |  |
| LF/HF ln | 0.7 (0.5 – 1.1) | 0.2 | | 1.0 (0.6 – 1.7) | | | 0.9 | |  |
| DC category 1 vs. 0 | 0.6 (0.2 – 2.7) | 0.5 | | 1.0 (0.2 – 4.3) | | | 0.9 | |  |
| DC category 2 vs. 0 | 0.6 (0.3 – 1.1) | 0.1 | | 0.8 (0.4 – 1.5) | | | 0.4 | |  |
| AC ms | 1.1 (1.0 – 1.3) | 0.2 | | 1.0 (0.9 – 1.2) | | | 0.6 | |  |
| Similar to the primary analysis, the cardiovascular mortality multivariate^#^ model included the respective heart rate variability parameter, and diabetes mellitus, age, creatinine, history of myocardial infarction, peripheral arteriovascular disease. The all-cause mortality multivariate* model additionally included high-sensitivity CRP.  Abbreviations: HRVI, HRV triangular index: number of NN intervals over the number of NN-intervals in the modal bin; SDNN, standard deviation of all NN intervals; SDANN, standard deviation of the averages of NN intervals in all 5-min segments of 24 h recording; TINN, triangular interpolation of NN interval histogram; RMSSD, square root of the mean square of differences between adjacent NN intervals; PNN50, adjacent NN intervals differing > 50 ms in total NN intervals; ULF, ultra-low frequency; VLF, very low frequency; LF, low frequence; HF, high frequency; LF/HF, LF to HF ratio; DC, deceleration capacity; AC, acceleration capacity; CI, confidence interval. | | | | | | | | | |
